# Early Prediction and Outcome of Septic Encephalopathy in Acute Stroke Patients With Nosocomial Coma

**DOI:** 10.14740/jocmr2176w

**Published:** 2015-05-08

**Authors:** Dao-Ming Tong, Ye-Ting Zhou, Guang-Sheng Wang, Xiao-Dong Chen, Tong-Hui Yang

**Affiliations:** a Department of Neurology, Affiliated Shuyang People’s Hospital, XuZhou Medical College, Jiangsu, China; b Department of Neurology, the Affiliated Pingxiang Hospital, Southern Medical University, China; c Medical Evaluation Unit, Affiliated Shuyang People’s Hospital, XuZhou Medical College, Jiangsu, China; d These authors contributed equally to this work and shared first authorship.

**Keywords:** Stroke, Infection, Septic encephalopathy, Systemic inflammatory response syndrome, Nosocomial coma, Outcome

## Abstract

**Background:**

Septic encephalopathy (SE) is the most common acute encephalopathy in ICU; however, little attention has been focused on risk of SE in the course of acute stroke. Our aim is to investigate the early prediction and outcome of SE in stroke patients with nosocomial coma (NC).

**Methods:**

A retrospective cohort study was conducted in an ICU of the tertiary teaching hospital in China from January 2006 to December 2009. Ninety-four acute stroke patients with NC were grouped according to with or without SE. Risk factors for patients with SE were compared with those without SE by univariate and multivariate analysis.

**Results:**

Of 94 stroke patients with NC, 46 (49%) had NC with SE and 48 (51%) had NC without SE. The onset-to-NC time was significant later in stroke patients with SE than those without SE (P < 0.01). There was a significant difference in body temperature, heart rate, respiratory rate, white blood cell (WBC), systolic blood pressure (SBP), diastolic blood pressure (DBP), systemic inflammatory response syndrome (SIRS), acute respiratory failure, septic shock, hypernatremia, and sequential organ failure assessment (SOFA) score between the SE and non-SE group (P < 0.05). On a repeat head imaging, vasogenic edema (P = 0.023) and subcortical white matter lesions (P = 0.011) were significantly higher in patients with SE than those without SE, while hematoma growth (P = 0.000), infarction progress (P = 0.003), and recurrent subarachnoid hemorrhage (SAH) (P = 0.011) were significantly lower in patients with SE than those without SE. Patients with SE had higher adjusted rates of fever ≥ 39 °C (odds ratio (OR): 2.753; 95% confidence interval (CI): 1.116 - 6.794; P = 0.028) and SIRS ≥ 3 items (OR: 6.459; 95% CI: 2.050 - 20.351; P = 0.001). The 30-day mortality in stroke patients with SE was higher than those without SE (76.1% vs. 45.8%, P = 0.003).

**Conclusion:**

High fever and severe SIRS are two early predictors of stroke patients with SE, and survival rates were worse in stroke patients with SE than those without SE.

## Introduction

Infection is a common cause of many systemic diseases or intracranial conditions associated with an impaired consciousness. Sepsis has a nationwide annual incidence of 750,000 and is defined as the host reaction to infection characterized by a systemic inflammatory response [1]. More and more observed showed many systemic infections in patients with different levels of consciousness (range from confusion to lethargy or coma), which has been known as septic encephalopathy (SE) or called “sepsis-associated encephalopathy (SAE)”. SE is the most common type of acute encephalopathy that is seen within varied ICU. The incidence of SE was 70% of septic patients [2, 3]. Its mortality rate was 40.5-71.9% [4], but whether the severity of a causal relationship remains uncertain [5]. The pathophysiology of SE is not yet clear, but the animal and human studies have shown that it may be attributed to the inflammatory cytokine, microcirculation failure, blood brain barrier (BBB) dysfunction, abnormal neurotransmitter, bacteria and/or endotoxin on directly affect the central nervous system (CNS) [6, 7]. Existing evidence is that SE is a solitary encephalopathy, or even that it is mutually exclusive with acute stroke diagnosis [8, 9]. Infection in stroke has aroused extensive clinical attention [10, 11]; however, few reports described the risk of sepsis in acute stroke [12]. Our research is to assess the risk factors and outcome of SE in acute stroke patients with nosocomial coma (NC).

## Methods

### Ethics statement

The study was approved by the Ethical Committee on Clinical Research of the Shuyang People’s Hospital, China. The study was in full compliance with the Helsinki Declaration, and solely required de-identification of all personal information related to the already collected clinical data without requirement of informed consent.

### Study setting and participants

This was a retrospective cohort study of all registered in an adult neurocritical care unit (NCCU, six critical care beds in total) of the tertiary teaching hospital in southern China between January 2006 and December 2009. Ninety-four acute stroke patients were grouped according to the NC with or without SE.

### Patients identified

Based on the International Statistical Classification of Diseases, 10th Revision (ICD-10) by WHO in 1994 discharged, we identified those patients who had acute cerebral stroke (code I60, I61, and I63). A total of 46 acute stroke patients with NC with SE (31 males and 15 females; range, 34 - 80 years) were identified as a patient group, and 48 acute stroke patients with NC without SE (30 males and 18 females; range, 30 - 84 years) were randomly selected as a control group.

A general diagnostic criterion for SE can be defined as a diffuse cerebral dysfunction induced by severe sepsis/severe systemic inflammatory response syndrome (SIRS), and without clinical or laboratory evidence of direct infectious involvement of the CNS [6, 7]. In this study, the SE in stroke patients was diagnosed using the following criteria: 1) NC resulting from a severe sepsis/severe SIRS with infection; no evidence of meningitis/encephalitis; 2) a repeat CT scan or MRI revealed vasogenic edema or white matter lesions, and coma cannot be explained by primary stroke; no evidence of significant intracerebral hemorrhage growth/infarction progressing/recurrent subarachnoid hemorrhage (SAH). Exclusion criteria of SE were as follows: 1) patients with SIRS but no infection; 2) present evidence of other non-SE. NC was defined as a coma that developed inside the hospital admission; if the coma developed earlier than this, it was considered to be acquired out-of-hospital. NC was diagnosed by using a Glasgow coma scale (GCS) score less than 8.

### Related definition

Significant intracerebral hemorrhage growth was defined as hematoma enlargement > 33% [13] and hematoma enlargement led to a cerebral midline shift/herniation. Infarction progressing was related to a new ischemic event or to the original stroke, such as stroke deterioration, evolution, extension, progression, or neurological damage or deterioration resulting from the original stroke. Rehemorrhage after the initial SAH was defined as a coma after sudden headache and new bleed was confirmed by head CT.

Sepsis is defined as presence of infection plus SIRS [14]. Severe sepsis was defined by the presence of two or more signs of SIRS resulting from a proven or suspected infectious process and at least one organ dysfunction associated with sepsis [14].

SIRS is defined as presence of two or more of the following: 1) temperature greater than 38 °C or less than 36 °C; 2) heart rate greater than 90 beats per minute; 3) tachypnea > 20 respirations per minute or PCO_2_ < 32 mm Hg; 4) white blood cell (WBC) count greater than 12.0 × 10^9^/L or less than 4.0 × 10^9^/L, or more than 10% band forms [14]. The severity of SIRS was assessed (0 = no anyone; 2 items = mild; ≥ 3 items = severe).

### Data collection

All patients underwent an initial CT scan on admission, and most patients had a repeat brain CT or MRI after NC onset. A neuroradiologist and an experienced neurologist evaluated all CT scan and MRI. The stroke location and type, risk factors of cerebrovascular, chest radiography and conditions of brain imaging were recorded. The following data and the laboratory measurements were recorded by an experienced neurologist: body temperature, blood pressure, heart rate, respiratory rate, creatinine, bilirubin, GCS score, sequential organ failure assessment (SOFA) score, serum glucose, sodium, potassium, WBC count, platelet count, and bacterial cultures. The onset-to-NC time, the length of ICU and hospital stay were also recorded. Baseline characteristics and outcomes at 30 days of follow-up were compared between these groups (SE or non-SE).

### Statistical methods

The results in each group were expressed as mean ± standard deviation (SD) or medians (IQR), and n (%) for qualitative values. Differences between patients were considered significant if the P-value was < 0.05. Patients with SE were compared with patients without SE by univariate analysis. Fisher’s exact test and Mann-Whitney U test were used to explore the relationship between baseline patient variables. Continuous variables were compared using the *t* test. Independent predictors of SE were identified with forward stepwise logistic regression. Survival analysis was performed using the Kaplan-Meier curve method. Statistical calculations were performed using a proprietary, computerized statistics package (SPSS 10.0).

## Results

The clinical baseline characteristics of the patients with and without SE are shown in Table 1. There was statistically significant (P < 0.005) onset-to-NC time in the SE group (medians, 3.7 days) than in the non-SE group (medians, 1.5 days). The hematoma volume and infarction volume were significantly lower in patients with SE than those without SE (P < 0.05).

**Table 1:** Characteristics of NC in Stroke Patients With and Without SE

Episodes	NC with SE (N = 46)	NC without SE (N = 48)	P
Female gender (%)	31/15	30/18	0.619
Age (years, mean ± SD)	61.5 ± 11.2	59.6 ± 12.5	0.431
Primary intracerebral hemorrhage (%)	24 (52.2)	27 (56.3)	0.692
Striato-capsula (%)	12 (50.0)	16 (59.3)	0.443
Lobar (%)	7 (29.2)	8 (29.6)	0.848
Thalamus (%)	2 (8.3)	2 (7.4)	0.088
Brain stem (%)	3 (12.5)	1 (3.7)	0.287
Intraventricular extension (%)	8 (33.3)	10 (37.0)	0.672
Hematoma volume (mL)	17.6 ± 12.9	31.6 ± 20.7	0.007
Infarction (%)	19 (41.3)	13 (27.1)	0.146
Cardioembolic stroke (%)	1 (5.3)	1 (7.7)	0.780
Atherothrombotic (%)	10 (52.6)	9 (69.2)	0.348
Lacunar stroke (%)	7 (36.8)	3 (23.1)	0.409
Other (%)	1 (5.3)	0 (0.0)	0.401
Cerebral infarcts volume (mL)	18.4 ± 63.5	78.9 ± 98.0	0.042
Subarachnoid hemorrhage (%)	3 (6.5)	8 (16.7)	0.126
Underlying diseases			
Hypertension (%)	23 (50.0)	31 (64.6)	0.153
Atrial fibrillation (%)	3 (6.5)	2 (4.2)	0.611
Diabetes (%)	2 (4.3)	5 (10.4)	0.263
Conditions of NNC			
Median ONCT (days, range)	3.7 (19.0)	1.5 (41.0)	0.002
Central brain herniation (%)	27 (58.7)	28 (58.3)	0.972
Uncal herniation (%)	5 (10.9)	9 (18.8)	0.283
Mechanical ventilation (%)	29 (63.0)	25 (52.1)	0.304
Length of ICU and hospital stay (days)	11.4 ± 12.7	11.9 ± 11.8	0.850
mRS score, mean ± SD	5.1 ± 1.6	4.9 ± 1.6	0.650
Mortality in 30 days (%)	35 (76.1)	22 (45.8)	0.003

NC: nosocomial coma; SE: septic encephalopathy; ONCT: onset-to-NC time; mRS: Rankin scale.

Forty-six SE patients with severe SIRS were diagnosed. Of those patients with severe SIRS, 20 (43.5%) patients had a pulmonary infection, and 15 (32.6%) had a bloodstream infection, followed by tracheobronchial, urinary tract, and other. No differences were observed in the frequency of the infection onset between patients with ICU-acquired or community-acquired infection. A total of 35 of 46 (76%) patients with severe SIRS had positive organisms isolated by organ/tissue culture, but blood cultures were microbiologically confirmed in minority of the clinical sepsis (15/46, 32.6%).

The results of univariate analysis and repeat head imaging are shown in Table 2. There was no difference in platelet count, initial GCS score, acute renal failure, hyperglycemia, hyponatremia, acute hepatic failure, acute seizures, diffuse cerebral edema, thalamus compression/central herniation, midbrain compression/uncal herniation, and hemorrhagic transformation among patients with SE and non-SE (P > 0.05). The presence of SE was significantly associated with higher temperature, faster pulse rate, faster respiratory rate, elevated WBC, high SBP, normal DBP, severe SIRS, acute respiratory failure, septic shock, hypernatremia, and high SOFA score.

**Table 2:** Univariate Analyses of NC in Stroke Patients With and Without SE

Episodes	NC with SE (N = 46)	NC without SE (N = 48)	P
Body temperature ≥ 39 °C (%)	34 (73.9)	3 (6.3)	0.000
Heart rate (beats/min)	109.5 ± 16.7	88.5 ± 19.9	0.000
Respiratory rate (breaths/min)	27.9 ± 6.4	22.5 ± 5.1	0.000
leukocyte count (× 10^9^/L)	14.9 ± 4.6	11.2 ± 6.0	0.000
Platelet count (× 10^9^/L)	220.3 ± 40.3	219.9 ± 30.6	0.966
SBP, mm Hg, mean ± SD	148.3 ± 47.3	168.6 ± 46.9	0.039
DBP, mm Hg, mean ± SD	81.6 ± 25.8	99.8 ± 30.9	0.023
Initial GCS, mean ± SD	10.2 ± 1.4	10.1 ± 1.4	0.894
Severe SIRS (%)	46 (100.0)	3 (6.3)*	0.000
Acute respiratory failure (%)	18 (39.1)	6 (12.5)	0.003
Septic shock (%)	8 (17.4)	0 (0.0)	0.003
Acute renal failure (%)	12 (26.1)	8 (16.7)	0.265
Acute hepatic failure (%)	2 (4.3)	1 (2.0)	0.532
Hyperglycemia, mean ± SD	6.55 ± 2.34	6.81 ± 2.91	0.685
Hypernatremia (> 150 mmo1/L)	7 (15.2)	1 (2.0)	0.016
Hyponatremia (< 110 mmol/L)	0 (0.0)	1 (2.0)	0.325
Acute seizures (%)	3 (6.5)	4 (8.3)	0.738
SOFA, mean ± SD	4.8 ± 2.7	2.5 ± 1.9	0.000
Repeat CT scan or MRI			
Vasogenic brain edema (%)	24 (52.2)	6 (12.5)	0.000
Subcortical whiter matter lesion (%)	10 (21.7)	2 (4.2)	0.011
Diffuse cerebral swelling (%)	32 (69.6)	40 (83.3)	0.115
Thalamus compression/central herniation (%)	33 (71.7)	32 (66.7)	0.595
Midbrain compression/uncal herniation (%)	5 (10.9)	9 (18.8)	0.238
Hematoma growth (%)	1 (2.2)	23 (47.9)	0.000
Infarction progress (%)	2 (4.3)	11 (22.9)	0.009
Recurrent SAH (%)	0 (0.0)	6 (12.5)	0.011
Hemorrhagic transformation (%)	0 (0.0)	2 (4.2)	0.162

NC: nosocomial coma; SE: septic encephalopathy; SBP: systolic blood pressure; DBP: diastolic blood pressure; GCS: Glasgow coma scale; SOFA: sequential organ failure assessment. *The patients with SIRS without infection.

On repeat imaging, of 46 stroke patients with SE, vasogenic edema around the stroke site was in 21 cases, and subcortical white matter lesions were in 10 patients. Vasogenic edema and subcortical white matter lesions were significantly higher in patients with SE than those without SE, but hematoma growth, infarction progress, and recurrent SAH were significantly lower in patients with SE than those without SE.

Table 3 summarizes the results of multiple logistic regressions based on our data of the original variables. This analysis demonstrated that fever ≥ 39 °C (P < 0.05) and SIRS ≥ 3 items (P < 0.01) were significantly and independently related to SE in acute stroke patients with NC.

**Table 3:** Multivariate Odds Ratios for SE in Acute Stroke

Episodes	OR	95% CI	P
Fever (≥ 39 °C)	2.753	1.116 - 6.794	0.028
SIRS (≥ 3 items)	6.459	2.050 - 20.351	0.001

SE: septic encephalopathy; SIRS: systemic inflammatory response syndrome; OR: odds ratio; CI: confidence intervals.

**Figure 1:**
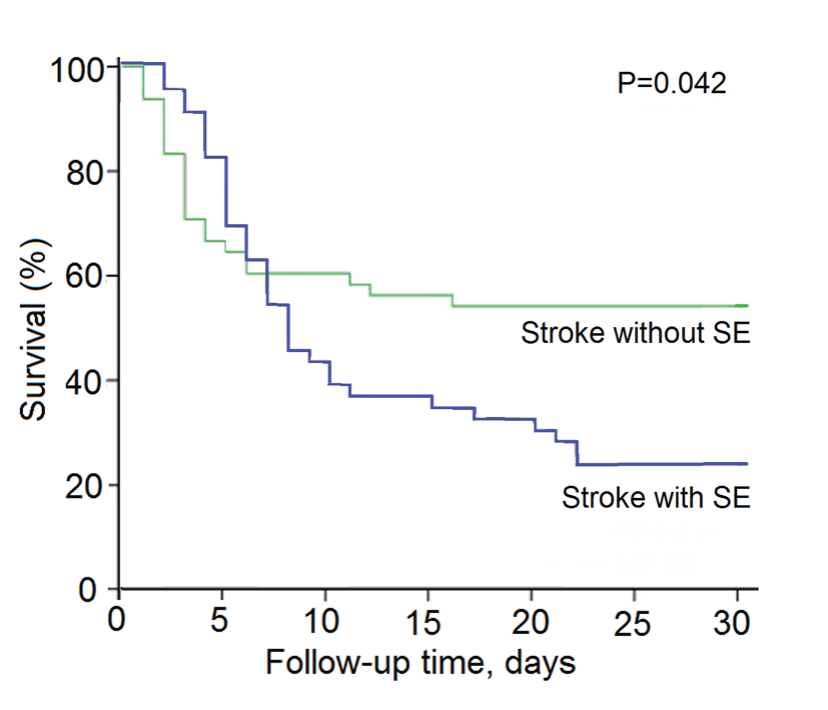
Kaplan-Meier survival curves for acute stroke patients with nosocomal coma with or without SE.

During 30 days follow-up, survival data were available for stroke patients with SE or without SE. Estimated unadjusted mortality of stroke patients with SE was significantly higher than those without SE (76.1% vs. 45.8%, P = 0.003). The unadjusted hazard ratio for survival in the stroke patients with SE than those without SE was 4.1 (95% confidence interval (CI): 13.47 - 18.36; P = 0.042) (Fig. 1).

## Discussion

Previous studies have confirmed that SE accounts for 70% of septic patients [2, 3]. SE was initially noted to be a monophasic event that should be excluded from acute stroke [8, 9]. Several studies have demonstrated that the prevalence of infection among patients with stroke was high [10, 11] and that the sepsis in stroke patients was not infrequent [12, 15, 16].

Previous studies have shown that imaging confirmed stroke progress mainly occurs within 6 - 24 h from symptoms onset [17, 18], but our study demonstrated that the median onset-to-NC time of acute stroke with SE was 3.7 days. Therefore, according to the results of the present study, the patients with stroke with SE who underwent repeat imaging after NC should be considered sufficient to exclude significant hematoma growth, infarction progress, and recurrent SAH. The present data showed that the SE patients have several risk factors: fever, increased WBC, severe SIRS, SOFA score > 4.8, acute respiratory failure, septic shock, vasogenic brain edema, and white matter lesions. Multiple logistic regression analyses based on the data of the original risk factors demonstrated that only fever and severe SIRS were significantly and independently related to SE in acute stroke with NC.

Fever is the most frequent manifestation that elicits suspicion of sepsis [19]. Neurotransmitter release, increased levels of oxygen radicals, destabilized cell membranes, and increased numbers of abnormal electrical depolarization have been imposed in fever process [20]. Even a small increase in temperature of 1.0 °C can produce a brain injury [21], and hyperthermia worsens injury even if it occurs 24 h after the original insult [22]. However, the SIRS also includes the features other than fever. The fact that fever and SIRS may be an independent predictor for neurologic deterioration has been demonstrated [23]. The present study indicated a high prevalence of fever and severe SIRS in preceding NC in stroke patients, which leads to almost two- to six-fold of the SE in stroke patients with NC than those without NC. In other words, fever and severe SIRS should be two high risk factors for SE in stroke patients.

Infections that present subsequent to stroke also were found to have complicated up to 41.5% of cases of stroke in ICU [8]. The present study showed that fever and severe SIRS are two frequent events due to infection in acute stroke patients with NC with SE, of those from pulmonary accounted for 43.5% and blood in 28.3%. Although a positive blood-based microbiological testing may not be observed [12], or may not be identified in 30-50% of patients that demonstrate a septic clinical presentation [24], a repeat brain CT scan or MRI for patients with stroke with NC is very important. Our study demonstrated that vasogenic edema and white matter lesions were significantly higher in patients with SE than those without SE.

Although vasogenic edema may also be present in 1 - 14 days and later after intracerebral hemorrhage [25], inflammatory mechanisms may play a crucial role in development of vasogenic edema, and inflammatory cells and mediators are important confounding factors in ischemic brain injury [26]. Thus, subcortical white matter lesions and microinfarctions may also occur. At least some alterations in the inflammatory response can cause cytokines and endothelial activation, leukocyte infiltration, and further tissue injury [26, 27]. The finding of this study could explain that fever and inflammatory responses might generate BBB collapse. We think, therefore, that vasogenic edema progression or white matter lesions after stroke might represent an inflammatory responses-induced SE.

The mortality rates (76.1%) of stroke patients with SE in the current study were higher than that in previous studies [4, 9, 11]. Several findings can explain the difference. First, the severity of late brain injury might be different. For example, vasogenic edema in patients with SE was a more frequent event than those without SE. Second, the present study showed that the SOFA score in the SE group was significantly higher than that in the non-SE group, whereas previous studies also suggested that the mortality of SE is almost always due to multiple organ failure [28]. Third, the cases of this study were only involved severe SE (all of NC). Therefore, the poor prognosis of stroke patients with SE with NC is more likely multifactorial, such as the severity of vasogenic edema, presence of high SOFA score, and other complications.

Some limitations should be considered in the present study. First, those comatose patients with stroke with SE in pre-hospital were excluded, which may underestimate the rate of SE. Second, the study shares the limitations of all retrospective studies, particularly in that the plan of follow-up MRI was low. In addition, the data were not collected for the primary purpose of identifying minor SE, which may overestimate the mortality of SE.

In conclusion, vasogenic edema or white matte lesion resulting from infection is a common clinical feature in stroke patients with SE. A fever ≥ 39 °C or SIRS ≥ 3 items emerged as the most powerful predictor of SE after acute stroke, and survival rates were worse in stroke patients with SE than those without SE. This information may be important in stroke patients with SE.
